# Left Pulmonary Vein Trunk Length as a Robust Predictor of Long-Term Success of Atrial Fibrillation Catheter Ablation

**DOI:** 10.31083/j.rcm2508301

**Published:** 2024-08-22

**Authors:** Jiaju Li, Zhe Wang, Fen Qin, Fangyuan Luo, Jiawei Chen, Yankun Liu, Hailong Tao, Jianzeng Dong

**Affiliations:** ^1^Department of Cardiology, The First Affiliated Hospital of Zhengzhou University, 450052 Zhengzhou, Henan, China; ^2^Department of Cardiology, China-Japan Friendship Hospital, 100029 Beijing, China; ^3^Graduate School of Peking Union Medical College, Chinese Academy of Medical Science, 100029 Beijing, China; ^4^Department of Cardiology, Beijing Anzhen Hospital, Capital Medical University, 100029 Beijing, China

**Keywords:** atrial fibrillation, catheter ablation, pulmonary vein anatomy, computed tomography

## Abstract

**Background:**

Radiofrequency catheter ablation (RFCA) is a commonly used treatment for atrial fibrillation (AF), but the long-term recurrence rate remains relatively high. Given the inconsistent results regarding the role of left pulmonary vein (PV) ostial anatomy in post-ablative recurrence of RFCA in previous studies, we sought to investigate the role of left PV trunk length using an alternative methodology.

**Methods:**

A total of 369 AF patients undergoing catheter ablation were included. The left/right trunk length (LTL/RTL) of the PV was measured from pre-ablative computed tomography (CT) using three-dimensional reconstruction techniques. We constructed three multivariable Cox models, with the inclusion of the LTL, RTL, and no LTL/RTL, and used the Delong test, integrated discrimination index (IDI), and net reclassification index (NRI) to assess model improvement. We identified optimal cut-off values for LTL with the receiver operating characteristic (ROC) curve, and estimated outcomes using the Kaplan-Meier survival curve. We also used subgroup analysis to evaluate interactions.

**Results:**

The results of the Delong test, IDI, and NRI indicated that LTL had a favorable impact on the performance of the multivariate model. Subsequently, the multivariate Cox regression analysis identified LTL as a significant risk factor for post-ablative recurrence of AF (adjusted hazard ratio (HR) = 1.08, 95% CI: 1.05–1.12, *p* < 0.001). According to the ROC curve, the optimal cut-off value for LTL is 11.15 mm, and the Kaplan-Meier estimator revealed different outcomes (*p* < 0.001). We calculated *p* for interaction between LTL and other factors, and no significant interaction terms were observed.

**Conclusions:**

LTL is a robust prognostic indicator for post-ablative outcome in AF patients receiving RFCA, with a longer LTL indicating a higher risk of recurrence.

## 1. Introduction

Radiofrequency catheter ablation (RFCA) is a commonly utilized treatment modality for atrial fibrillation (AF), due to its ability to effectively restore sinus rhythm and alleviate symptoms [1]. Currently, first-line catheter ablation for AF has received a Class IIa recommendation for treating symptomatic patients with paroxysmal AF, and a Class IIb recommendation for those with persistent AF [2]. However, despite its efficacy, the long-term recurrence rate of AF after RFCA remains relatively high [3]. Therefore, preoperative assessment of patient risk factors is regularly undertaken by cardiac electrophysiologists, in order to provide guidance on specific treatment options and ultimately achieve better clinical outcomes [4].

Circumferential pulmonary vein isolation (CPVI) is the cornerstone of RFCA therapy [5]. Variant pulmonary vein (PV) anatomy is thought to be associated with a worse prognosis compared to normal PV anatomy. Previous studies had found that 8.5%–83% of patients experience a left common PV trunk (or common PV ostium) [6, 7, 8, 9, 10, 11, 12, 13]. In most studies, a common trunk refers to the length between the virtual border of the left atrium (LA) and the bifurcation of ipsilateral PVs that is >5 mm. However, previous studies found inconsistent results regarding the prognostic impact of this variant [6, 7, 8, 9, 10, 11, 12, 13]. Despite the potential clinical implication of a left common PV trunk, this variation only occurs in a subset of patients, and is extremely variable [14]. Categorizing cases that fall between the common and non-common trunk can often be challenging. Therefore, we sought to assess the role of PV trunk length, which is universally applicable to most patients. To achieve this, we defined a new measurement that reflects PV trunk length, and analyzed its role in long-term outcomes of AF patients undergoing catheter ablation.

## 2. Methods

### 2.1 Study Population

**Fig. 1.**
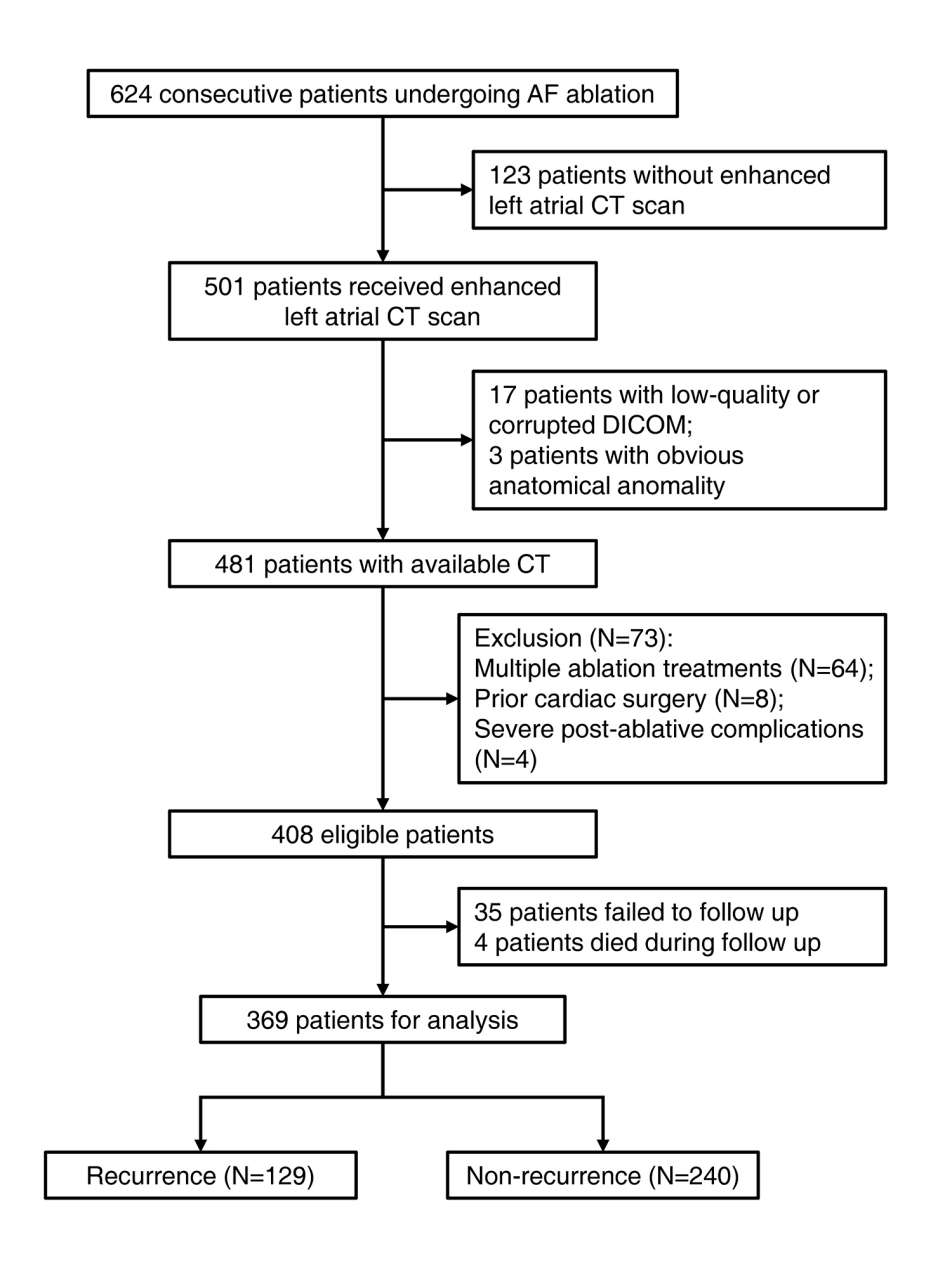
**The flow chart of the study**. AF, atrial fibrillation; CT, computed tomography; DICOM, digital imaging and communications in medicine.

This study included AF patients undergoing catheter ablation in the First Affiliated Hospital of Zhengzhou University between 2019 and 2021. All patients had undergone ablation assessment and perioperative management according to the European Society of Cardiology guidelines [2]. Demographic, clinical, and imaging data were collected and adhered to predefined inclusion criteria: (i) patients who were receiving their first RFCA treatment; (ii) patients with an available LA computed tomography (CT) scan within five days prior to RFCA; (iii) no prior heart surgery; (iv) no obvious anatomical anomalies in PVs (conjoined bilateral inferior PV; accessory PV that is inserted into LA); (v) no severe post-ablative complications. The study protocol adhered to the principles outlined in the Declaration of Helsinki and received approval from the local ethical review board. Informed consent was obtained from all participants. The study design was presented in Fig. 1.

### 2.2 CT Measurement

**Fig. 2.**
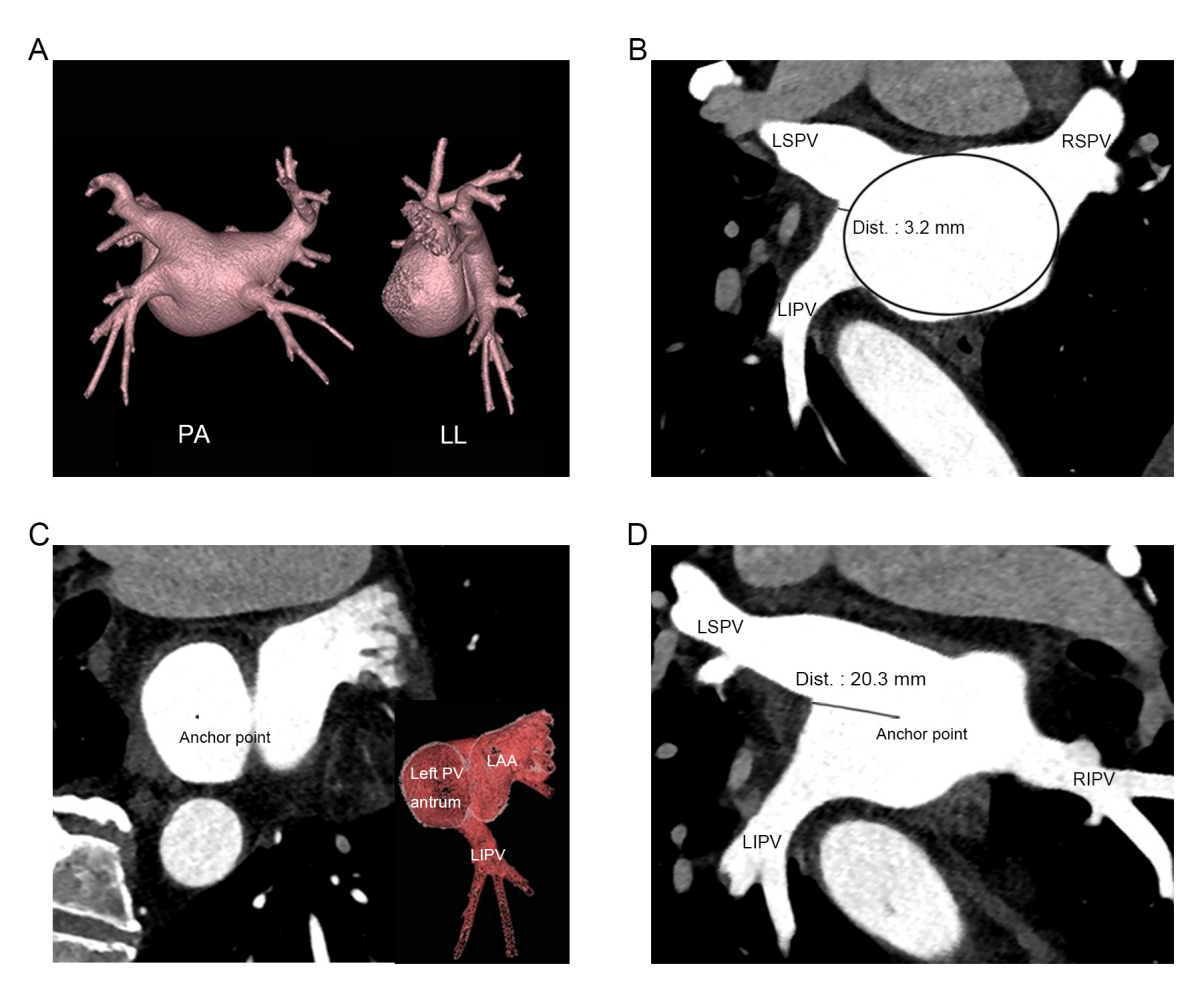
**Measurement and comparison between the previous approach and the new approach**. (A) In a case where LA was visualized using three-dimensional reconstruction, two approaches were employed, respectively. (B) In the approach adopted by previous studies, the LA was considered as an ellipse, and the distance from the bifurcation to the boundary at the virtual border was measured. In this case, a length of 3.2 mm was obtained. (C) In the new approach, to measure left trunk length, first, locate the conjunction between PV antrum and LA in three-dimensional oblique view, identify the plane of PV antrum ostium and place the anchor point at its center. (D) Then, rotate the perspective and measure the length from anchor point to the ipsilateral intravenous ridge, resulting in a measured left trunk length of 20.3 mm. PA, posteroanterior; LL, left lateral; LA, left atrium; PV, pulmonary vein; Dist., distance; LAA, left atrial appendage; LSPV, left superior pulmonary vein; LIPV, left inferior pulmonary vein; RSPV, right superior pulmonary vein; RIPV, right inferior pulmonary vein.

All patients underwent a preoperative enhanced CT scan utilizing a 256-slice scanner (Revolution CT scanner, GE Healthcare, Munich, Germany). The scan was performed using a single breath-hold technique with prospective electrocardiogram (ECG) gating. During the scan, 35–60 mL of contrast agent (Iohexol 350 mgI/mL, Yangtze River Pharmaceutical Group, Taizhou, China) was administered at a rate of 3.5–6 mL/s. After LA reconstruction on a post-processing workstation (AW VolumeShare7, GE Healthcare, Munich, Germany), LA anteroposterior diameter, LA transverse diameter, LA longitudinal diameter, LA volume, left atrial appendage volume, left trunk length (LTL) and right trunk length (RTL) were measured. We regard the junction between the PV antrum and the LA as an ostium, which exhibits an obvious angulation on the surface of the LA. After mapping the antrum ostium, the center was automatically generated and the distance from the center to the ipsilateral PV intravenous ridge was defined as the left/right trunk length, which was measured in the three-dimensional oblique view, as shown in Fig. 2. Compared to the previous method, this new approach yields completely distinct results that were not affected by the shape or size of the LA. To ensure accuracy, the personnel involved in the CT measurements were blinded from the clinical data. The personnel were separated into two groups for measuring the parameters. If the measured parameters showed discrepancies over 5%, they were remeasured following a discussion.

### 2.3 Catheter Ablation

All patients received regular anticoagulation medication before catheter ablation and LA thrombosis was excluded by enhanced CT or transesophageal echocardiography. The ablation was performed with a 3.5 mm contact force-sensing radiofrequency catheter (Thermo-Cool SmartTouch Catheter, Biosense Webster Inc, Irvine, CA, USA) using a three-dimensional cardiac mapping system (CARTO 3, Biosense Webster Inc, Irvine, CA, USA). For patients with paroxysmal AF, bilateral CPVI was performed, while for those with persistent AF, the ‘2C3L’ approach involving bilateral CPVI and linear ablation for LA roof, mitral isthmus and tricuspid isthmus was applied [15]. Early reconnection sites were additionally ablated after a waiting phase of 30 minutes. If AF was sustained despite successful verification of isolation at each ablation site, an extra electrical cardioversion was performed. All patients were prescribed oral amiodarone for three months after the ablation. The antiarrhythmic medication was stopped if no recurrence was observed at the end of three months.

### 2.4 Follow-up

A one-year follow-up was conducted after the ablation, with scheduled outpatient visits every three months. ECG and 24-hour Holter monitoring were performed at each appointment. If symptoms of AF occurred between visits, ECG, and 24-hour Holter monitoring were performed to check for recurrence. The endpoint was defined as any recorded atrial arrhythmia over 30 seconds; the first three months after the ablation were considered as a blanking period.

### 2.5 Statistics

Continuous variables were presented as median (interquartile ranges), and categorical variables were shown as frequencies (percentages). Group comparisons were made using Mann-Whitney U test for continuous variables, and chi-squared test or Fisher’s exact probability test for categorical variables, as appropriate. Multivariate Cox models were constructed by including age, body mass index (BMI), and variables with significant between-group differences, after excluding significantly correlated variables determined by correlation analysis (Pearson R >0.60 was defined as significantly correlated). The performance improvement of the multivariate models was implemented with the Delong test, which compared the area under the receiver operating characteristic (ROC) curve. The model improvement was also evaluated using the integrated discrimination index (IDI) and the net reclassification index (NRI) [16]. Patients were stratified into two groups based on the inflection point of the LTL’s ROC curve, and Kaplan-Meier survival analysis curves were constructed. Differences between groups were compared using log-rank testing. Then, subgroup analysis was performed, and the hazard ratio (HR) of LTL were analyzed using univariate Cox analysis in each group. *p* values for interaction between LTL and age, BMI, LA anteroposterior diameter, LA volume, AF type, and whether AF recurred over three years were calculated to assess any interaction terms. Statistical significance was defined as *p* < 0.05 (two-tailed), and all statistical analyses were conducted using R (version 4.1.2, R Project Team, https://www.r-project.org).

## 3. Results

### 3.1 Baseline Characteristics

A total of 369 cases were included in our study, with a median age of 61 years. The median BMI was 25.8 kg/m^2^, 219 (59.3%) patients were male, 196 (53.12%) patients were diagnosed with paroxysmal AF and 173 (46.88%) patients with persistent AF. Based on whether there was recurrence within one year after ablation, the patients were divided into the recurrence or non-recurrence groups, as described in Table 1. The proportion of persistent AF was significantly higher in the recurrence group (*p* < 0.001), compared with the non-recurrence group. LA anteroposterior diameter, LA transverse diameter, LA volume, LTL, and RTL were larger in the recurrence group (*p* < 0.001, *p* = 0.003, *p* < 0.001, *p* < 0.001, and *p* = 0.001, respectively).

**Table 1.** **Baseline characteristics**.

Characteristics		Total (N = 369)	Recurrence (N = 129)	Non–recurrence (N = 240)	*p*
Age		61 (54–68)	62 (56–69)	59.5 (52–68)	0.063
Gender					0.749
	Male	219 (59.3%)	78 (60.5%)	141 (58.75%)	
	Female	150 (40.7%)	51 (39.5%)	99 (41.25%)	
BMI (kg/m^2^)		25.8 (23.9–28.1)	26.6 (24.2–28.3)	25.33 (23.6–28.1)	0.056
Persistent AF		173 (46.9%)	88 (68.2%)	85 (35.4%)	<0.001
AF time since occur (months)		24 (4–72)	36 (6–96)	24 (3–60)	0.170
Smoke		103 (27.9%)	34 (26.4%)	69 (28.7%)	0.625
Alcohol		75 (20.3%)	26 (20.2%)	49 (20.4%)	0.953
Hypertension		201 (54.5%)	76 (58.9%)	125 (52.1%)	0.209
Diabetes mellitus		81 (22%)	33 (25.6%)	48 (20%)	0.217
Coronary disease		99 (26.8%)	36 (27.9%)	63 (26.3%)	0.732
Hyperlipidemia		37 (10%)	15 (11.6%)	22 (9.2%)	0.453
Stroke history		65 (17.6%)	21 (16.3%)	44 (18.3%)	0.621
Preoperative BNP (pg/mL)		383 (127–808.5)	548 (168.5–953)	342 (104.5–794.8)	0.110
Preoperative EF (%), ultrasound		63 (60–64)	63 (58–64)	63 (60–64)	0.316
HbA1c (%)		5.9 (5.6–6.4)	5.9 (5.6–6.4)	5.9 (5.6–6.5)	0.695
Serum creatinine (µmol/L)		72 (61.9–81.2)	72.8 (61–85.5)	72 (62.4–81)	0.323
Uric acid (µmol/L)		311 (246–379)	320 (253–394)	307 (244.3–378.5)	0.408
Alanine aminotransferase (U/L)		20 (14–30)	20 (14–29)	20.5 (14–30)	0.641
Aspartate aminotransferase (U/L)		21 (17–27)	22 (17–28)	21 (17.3–26)	0.680
Gamma-glutamyl transferase (U/L)		26 (16.5–53)	26 (15–51)	26 (18–54)	0.511
Albumin (g/L)		41.3 (39.4–44)	41.2 (39.4–43.7)	41.5 (39.3–44.6)	0.725
LA anteroposterior diameter (mm)		45.5 (39.2–51.6)	49.2 (43.7–55.3)	42.6 (37.1–48.9)	<0.001
LA transverse diameter (mm)		75.1 (68.8–81.4)	77.2 (71–83)	74.2 (67.6–81)	0.003
LA longitudinal diameter (mm)		58.8 (55.3–63.1)	59.4 (55.8–64.1)	58.3 (54.9–63)	0.196
Left trunk length (mm)		12.4 (9.5–15.2)	14.2 (11.7–16.6)	11.3 (8.5–14.1)	<0.001
Right trunk length (mm)		8.8 (7.1–11.1)	9.3 (8.1 –11.3)	8.6 (6.6–10.8)	0.001
LAA volume (mm^3^)		12.2 (9.3–15.9)	12.8 (9.5–16.7)	11.6 (9–15.5)	0.260
LA volume (mm^3^)		133.3 (107.9–166.1)	145.8 (120–177.4)	124.6 (102.4–162.2)	<0.001

BMI, body mass index; AF, atrial fibrillation; LA, left atrial; EF, ejection fraction; LAA, left atrial appendage; BNP, B-type natriuretic peptide; HbA1c, hemoglobin A1c.

### 3.2 LTL and Long-Term Outcomes

**Fig. 3.**
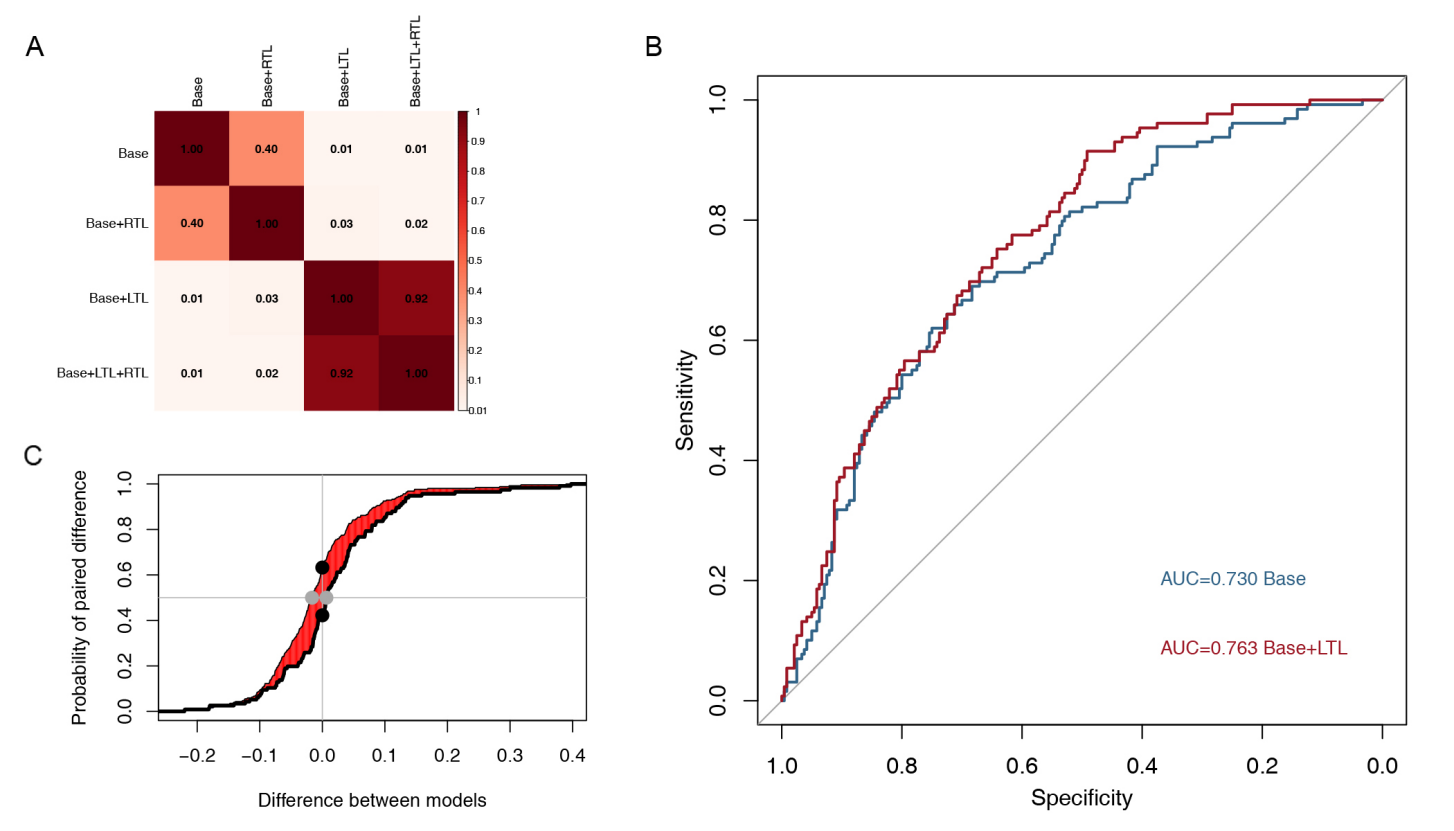
**Left trunk length optimizes the performance of the multivariate model**. (A) Results from the Delong test. Comparisons among four multivariate Cox models’ ROC curves were implemented and showed with *p* value. Inclusion of LTL significantly improves model performance (*p* < 0.05). (B) ROC curve of the base model and the base + LTL model. (C) Visualization of IDI and NRI between base model and base + LTL model. X-axis refers to the parameter ‘s’ according to Uno, H. *et al*. [16], Y-axis refers to the cumulated paired difference between the new model and the old model. The difference between the area under curves, colored in red, represents IDI; the distance between two black dots represents continuous NRI; and the distance between two grey dots represents median improvement in risk score. ROC, receiver operator characteristics; IDI, integrated discrimination index; NRI, net reclassification index; LTL, left trunk length; RTL, right trunk length; AUC, area under curve.

**Fig. 4.**
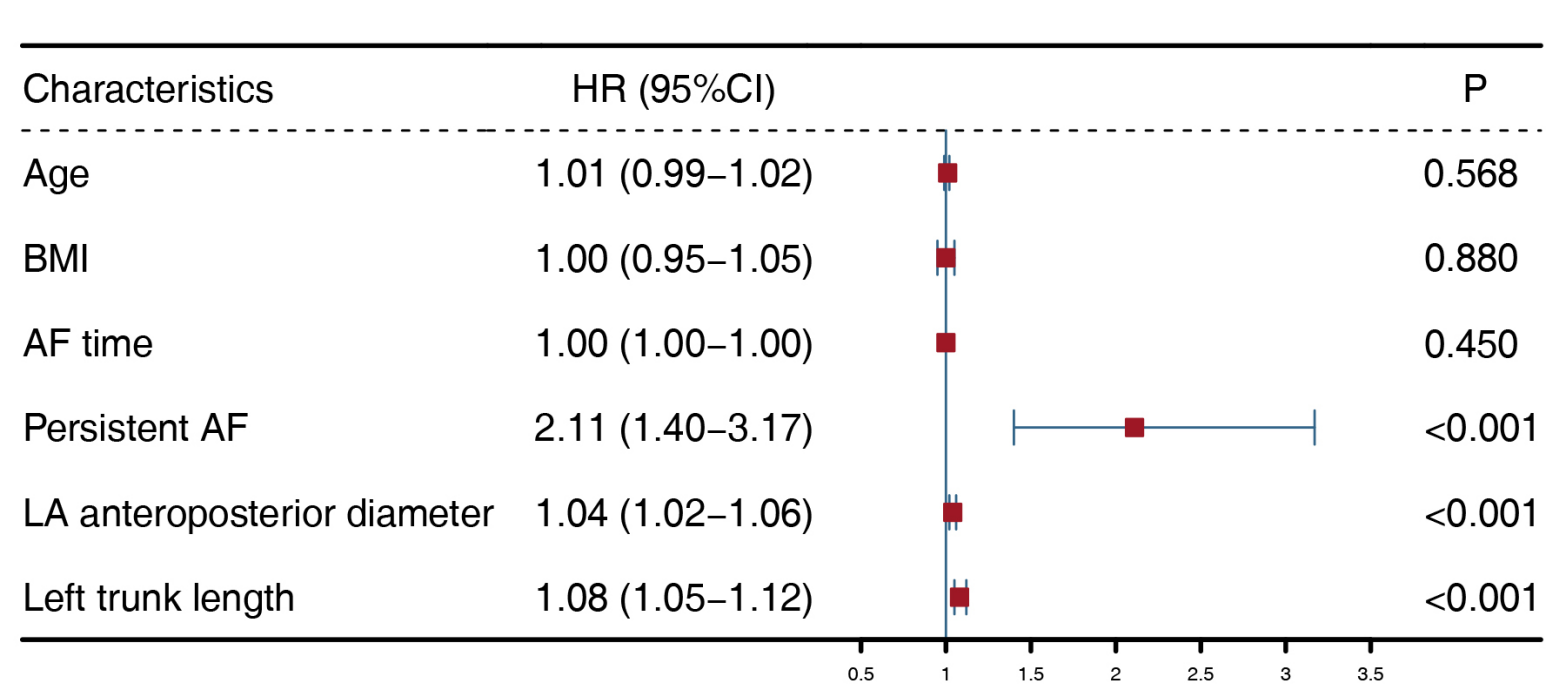
**Multivariate Cox model**. BMI, body mass index; AF, atrial fibrillation; LA, left atrial; HR, hazard ratio; CI, confidence interval.

**Fig. 5.**
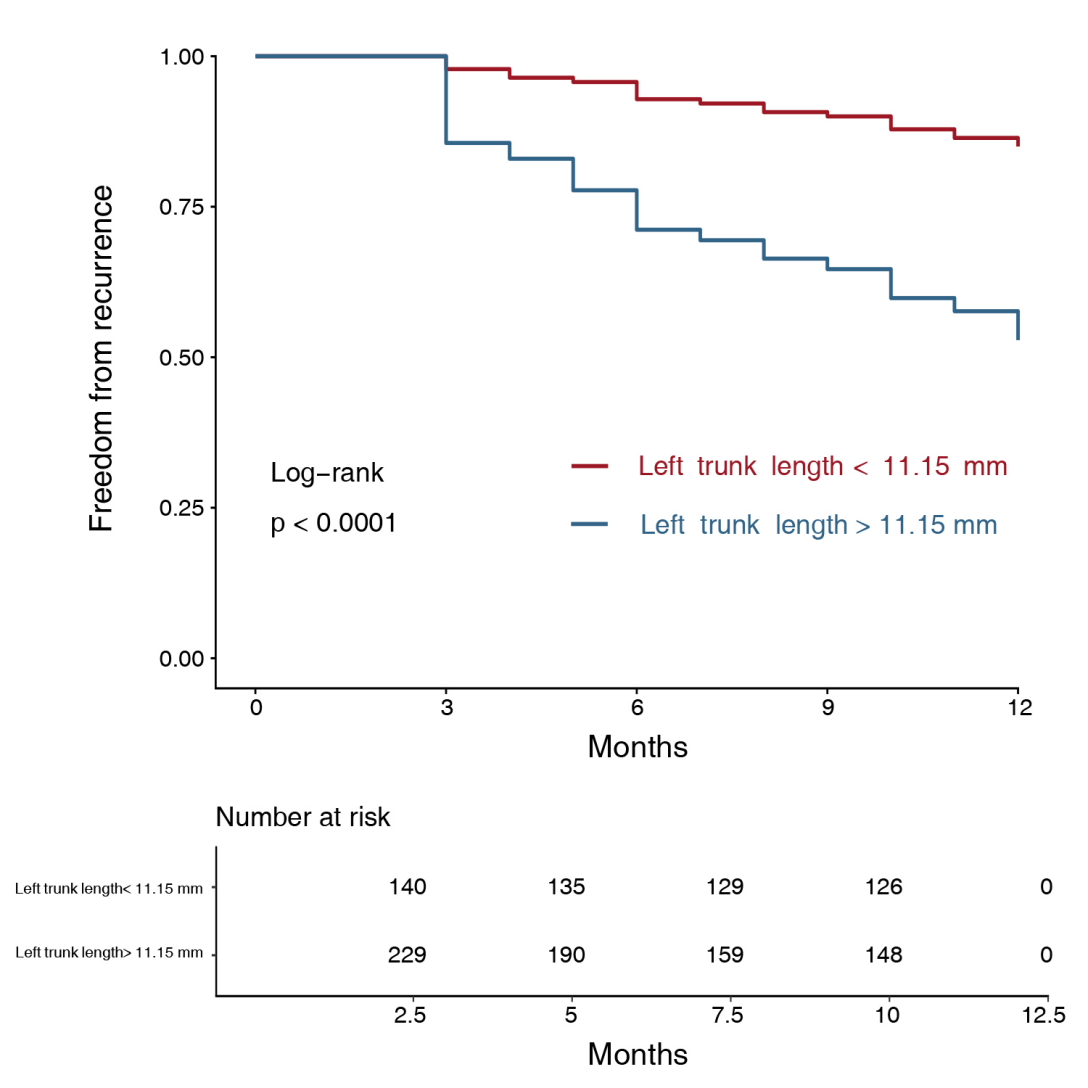
**Kaplan-Meier estimator based on left trunk length**. Patients with a longer left trunk length exhibits poorer outcome according to the Kaplan-Meier estimator (*p* < 0.001, log-rank method).

We included age, BMI, AF time, persistent AF, and LA anteroposterior diameter as variables in the multivariate Cox regression to build a base model, with the exclusion of LA volume and LA transverse diameter due to their significant correlation with LA anteroposterior diameter (Pearson R = 0.64 and 0.81, respectively). Three more models were generated based on whether LTL and RTL were added subsequently. The Delong test of the area under the ROC curve for the four models showed that adding LTL improved the model performance, while adding RTL did not affect the model performance (Fig. 3A, Ref. [16]). The ROC curves for the base model and base + LTL model are shown in Fig. 3B. We compared the IDI and NRI between the base model and the base added LTL model, and the results showed that adding LTL significantly increased the predictive ability of the model (Fig. 3C). Therefore, we ultimately selected age, BMI, AF time, persistent AF, LA posterior diameter, and LTL as the variables for the multivariate Cox regression. Among them, persistent AF (adjusted HR = 2.11, *p* < 0.001), LA anteroposterior diameter (adjusted HR = 1.04, *p* < 0.001), and LTL (adjusted HR = 1.08, *p* < 0.001) were risk factors for post-ablative AF recurrence (Fig. 4). The Kaplan-Meier estimator showed that there was a significant difference in freedom from recurrence between the two groups after grouping by the inflective cutoff value (LTL = 11.15 mm, sensitivity = 0.837, specificity = 0.496) of the ROC curve for LTL (Fig. 5).

### 3.3 The Impact of LTL on Different Subgroups

**Fig. 6.**
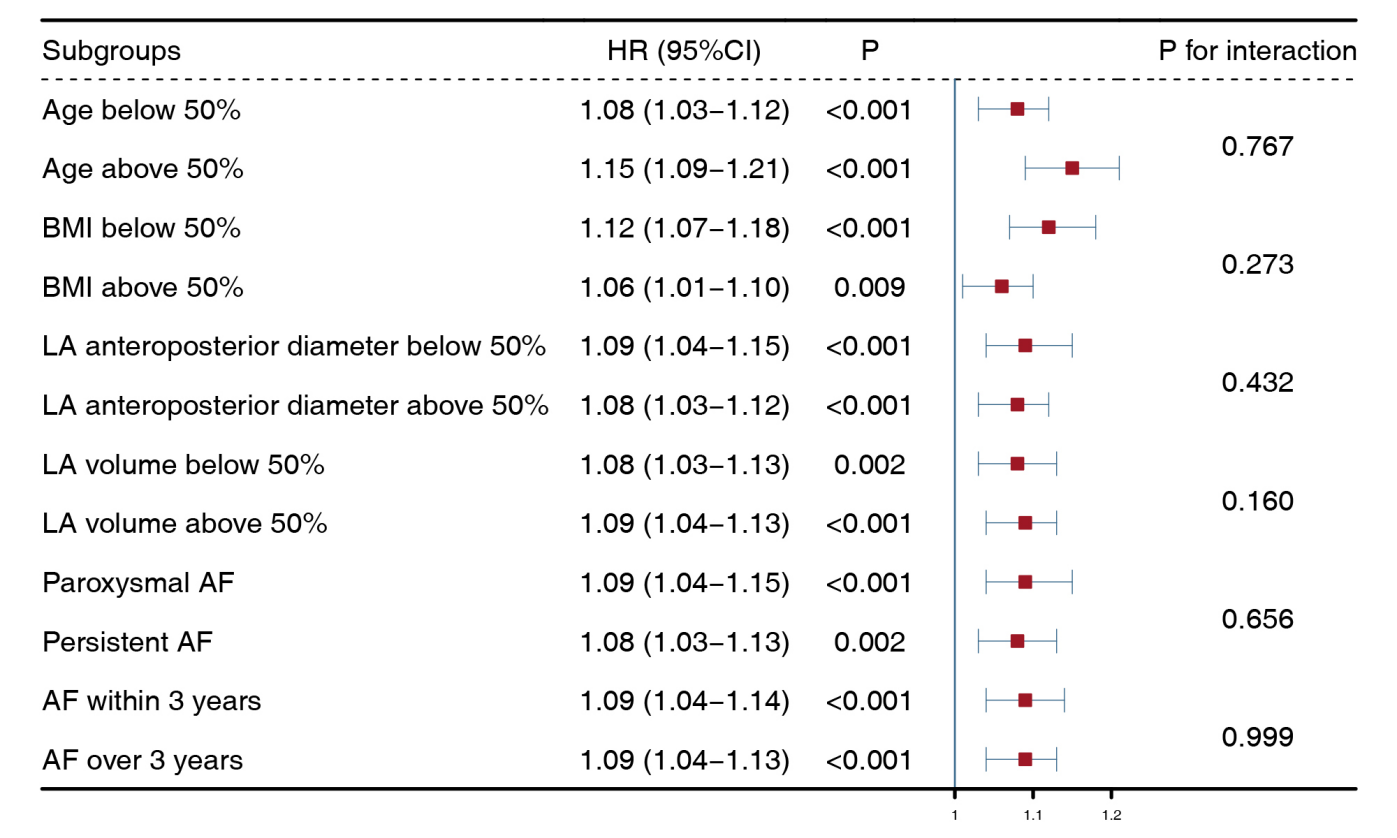
**Subgroup analysis of left trunk length**. Univariable Cox regression revealed significant prognostic value of left trunk length in all subgroups (*p* < 0.05, the third row). Additionally, no statistically significant interactions were observed (*p* > 0.05, the fifth row). This suggested that left trunk length had an independent impact on patients’ outcomes in all subgroups. BMI, body mass index; LA, left atrial; AF, atrial fibrillation; HR, hazard ratio; CI, confidence interval.

Patients were grouped according to the median age, BMI, LA anteroposterior diameter, and LA volume, as well as paroxysmal/persistent AF and AF over/within 3 years. Univariate Cox regression showed that LTL was a risk factor in all subgroups. We calculated the interaction *p* values for continuous variables between LTL with age, BMI, LA anteroposterior diameter, LA volume, as well as those for categorical variables between LTL and paroxysmal/persistent AF, AF over/within 3 years. As a result, no significant interactions were observed between LTL and these variables (Fig. 6).

## 4. Discussion

This was a single-center retrospective study in which we analyzed the role of LTL measured by ECG-gated enhanced CT in AF patients before RFCA in predicting recurrence within one year. To our knowledge, we were the first to define and evaluate the effect of LTL. Our study confirmed that LTL is an independent risk factor for post-ablative recurrence of RFCA. Currently, cardiac electrophysiologists recognize the significant impact of LA anatomy and its associated structures on RFCA outcomes for AF. LA size is one of the most frequently considered prognostic indicators before ablation [4]. Preoperative cardiac CT is commonly used in many centers since it can accurately reflect the anatomical structure of the LA and can exclude the presence of atrial thrombosis [4, 17, 18, 19]. Our research revealed new insight for guiding clinical practice and improving treatment outcomes, by showing that LTL is another independent, robust predictor for treatment outcomes.

In our study population, the baseline characteristics between the recurrence group and non-recurrence group were different in terms of AF type, AF time, LA size (including anteroposterior diameter, transverse diameter, and volume), and bilateral PV trunk length. With the exception of LTL and RTL, the role of these factors has been well-recognized [2, 20]. Although both LTL and RTL were different between the recurrence and non-recurrence group, the results of the DeLong test indicated that incorporating LTL with baseline characteristics significantly improved the model performance, whereas the inclusion of RTL did not. Therefore, we selected the Cox model that incorporates LTL. After adjusting for age, BMI, persistent AF, AF time, and LA anteroposterior diameter, the multivariate model showed that LTL was an independent risk factor for one-year recurrence. Additionally, the *p* value for interaction indicated no significant interaction between LTL and these factors, suggesting that the role of LTL in RFCA outcome was independent despite different AF types and ablation procedures, or LA size, age, and BMI.

The term left PV common trunk, or common ostium, refers to the length between the virtual border of the LA and the bifurcation of ipsilateral PVs that is >5 mm [7]. Early RFCA procedures performed deep within the PVs posed a potential risk for PV stenosis, which prompted the gradual shift of the ablation site to a location 5 mm away from the PV ostium [21]. We hypothesized that the standard value of 5 mm for determining the common ostium was established based on this observation. Nevertheless, the determination of the ‘LA virtual boundary’ can be influenced by various factors, especially LA sphericity and size [7]. Our findings suggested using a fixed structure as the boundary between the LA and the PV antrum for analysis.

The Impact of PV anatomy on ablative treatment for AF patients had been a long-standing research topic of considerable interest. Previous studies have investigated the influence of different patterns of PV variants on AF ablation, however, their findings have yielded inconsistent results. In a study by Hunter *et al*. [8] with 350 cases of AF, a common trunk of the left PVs was found to be associated with lower initial success rates and recurrence-free rates following RFCA. It is noteworthy that this study shared a similar RFCA procedural option with our study for paroxysmal and persistent AF [8]. Similarly, Sohns *et al*. [9] discovered that a left common trunk and other PV variants were linked to unfavorable outcomes after RFCA in AF patients. However, studies by Ströker* et al*. [10], Coutiño *et al*. [12], and Yorgun *et al*. [13] revealed no significant impact on the long-term outcomes of AF patients with a left common trunk after cryoballoon ablation. Coutiño* et al*. [12] also demonstrated that patients with longer or shorter common trunks had no significant difference in their long-term outcomes. Conversely, McLellan *et al*. [11] suggested that a left common trunk was a protective factor against AF recurrence after RFCA of paroxysmal AF. We reviewed these studies, and discovered that systemic bias may have been induced by these measurements. To address this issue, we proposed a fixed localization of the PV antrum orifice instead of using the virtual LA boundary; then we measured the distance from the ostium to the bifurcation of the PVs for further analysis. The results revealed a positive impact of LTL on long-term post-ablative recurrence. By evaluating LTL preoperatively, clinicians can make more informed decisions regarding RFCA for AF patients, conduct more rigorous post-ablative detection, and promptly manage early post-ablative recurrence, which may potentially improve patient outcomes.

## 5. Limitations

Our study had several limitations. First, this was a single-center retrospective study, with unavoidable biases. Second, in order to eliminate potential confounding factors, we excluded some extremely variated patterns of PV, including conjoined bilateral inferior PV and accessory PV that are inserted into the LA from the roof or posterior wall, but this may also have introduced new biases. Third, our center adopted the ‘2C3L’ ablation technique for persistent AF patients, while other centers may use different ablation procedures. Whether LTL had the same predictive value for long-term outcomes with other ablation techniques remains to be further studied. Despite these limitations, our study provides a perspective on the impact of left PV trunk anatomy for AF patients undergoing RFCA, and has identified LTL as a new robust indicator.

## 6. Conclusions

LTL is a robust prognostic indicator for post-ablative recurrence in AF patients receiving RFCA, with longer LTL indicating a higher risk of recurrence. Enhanced post-ablative monitoring and management for early recurrence can potentially improve the outcomes of these patients.

## Data Availability

The datasets used and analyzed during the current study are available from the corresponding author on reasonable request.
